# Orthodontic maxillary molar movement-induced zygomatic pillar remodeling and its consequences on occlusal characteristics and stress distribution

**DOI:** 10.1007/s00784-024-05713-3

**Published:** 2024-06-15

**Authors:** Size Li, Han Bao, Xiaojie Su, Liping Xiong, Qianwen Yin, Deao Gu, Leiying Miao, Chao Liu

**Affiliations:** 1 Department of Orthodontics, Affiliated Stomatological Hospital of Medical School, Nanjing Stomatological Hospital, Nanjing University, Nanjing, Jiangsu Province 210008 China; 2 Department of Endodontics, Affiliated Stomatological Hospital of Medical School, Nanjing Stomatological Hospital, Nanjing University, Nanjing, Jiangsu 210008 China

**Keywords:** Orthodontic treatment, Zygomatic pillar, Bone remodeling, The maxillary molar movement, The maximum occlusal force and occlusal contact area, Finite element analysis

## Abstract

**Objective:**

We aimed to evaluate changes in the zygomatic pillar during orthodontic treatment involving premolar extraction, analyze the effects of maxillary first molar movement on zygomatic pillar remodeling, and examine occlusal characteristics and stress distribution after remodeling.

**Methods:**

Twenty-five patients who underwent premolar extraction were included in the study. The zygomatic pillar measurement range was defined, and cross-sectional areas, surface landmark coordinates, alveolar and cortical bone thicknesses, and density changes were assessed using Mimics software based on the cone-beam computed tomography scans taken before (T0) and after the treatment (T1). Multiple linear regression analysis was performed to determine the correlation between changes in the zygomatic pillar and maxillary first molar three-dimensional (3D) movement and rotation. Additionally, the correlation between pillar remodeling and occlusal characteristics was analyzed by Teetester. Pre- and post-reconstruction 3D finite element models were constructed and loaded with an average occlusal force of two periods.

**Results:**

The morphological and structural remodeling of the zygomatic pillar after orthodontic treatment involving premolar extraction showed a decreased cross-sectional area of the lower segment of the zygomatic pillar. The zygomatic process point moved inward and backward, whereas the zygomatico-maxillary suture point moved backward. The thicknesses of the zygomatic pillar alveolar and cortical bones were thinner, and reduced alveolar bone density was observed. Simultaneously, the movement and angle change of the maxillary first molar could predict zygomatic pillar reconstruction to a certain extent. With decreasing the total occlusal force and the occlusal force of the first molar, occlusal force distribution was more uniform. With zygomatic pillar remodeling, occlusal stress distribution in the zygomatic alveolar ridge decreased, and occlusal stress was concentrated at the junction of the vertical and horizontal parts of the zygomatic bone and the posterior part of the zygomatic arch.

**Conclusions:**

Orthodontic treatment involving premolar extraction led to zygomatic pillar remodeling, making it more fragile than before and reducing the occlusal force of the maxillary first molar and the entire dentition with stress concentrated in weak areas.

**Clinical relevance:**

No other study has focused on the effects of orthodontics on pillar structures. The present study indicates that the mesial movement of the maxillary first molar weakened the zygomatic pillar and reduced occlusal function, thereby providing insights for inserting anchorage screws and facial esthetics.

## Introduction

Orthodontic treatment is based on a biological principle, which states that applying light forces to teeth for an extended period of time causes tooth movement accompanied by the remodeling of the surrounding bone [1]. Bone remodeling occurs in the cancellous and cortical bones during this process [2]. The alveolar bone extends upward to the maxillary bone, dispersing the applied forces throughout the skull. Sicher et al. proposed that three vertical pillars were formed, namely the canine pillar, zygomatic pillar, and pterygoid pillar, in the maxillary bone by its thickening aggregation along the direction of force transmission. These pillar structures, comprising the alveolar and cortical bones above, bear masticatory stress and external forces, providing optimal resistance to scaffolding [3]. During the orthodontic process, forces on the teeth are also transmitted along the pillars. However, existing studies on bone remodeling induced by orthodontic treatment have focused on examining changes in the alveolar bone, and a few studies have investigated whether orthodontic tooth movement is accompanied by the reconstruction of the pillar structures after they have been subjected to force.

Of the three pillars, the zygomatic pillar bears masticatory forces from the maxillary first molar originating from the alveolar bone and is bifurcated upwards via the zygomatic alveolar crest, one branch of which extends laterally from the orbital rim through the zygomatic process of the frontal bone to the skullcap, whereas the other branch extends posteriorly through the zygomatic arch to the skull base. The maxillary first molar plays a pivotal role in supporting occlusal forces as a critical component of the posterior dental support system [4, 5]; consequently, the zygomatic pillar is one of the most resistant areas in the craniofacial bones [6].

In orthodontic cases requiring premolar extraction, even with the maximum anchorage designed, the movement of posterior teeth accounts for approximately one-third of the extraction space [7]. Based on this phenomenon, ChoiDS et al. [8] performed three-dimensional (3D) finite element analysis (FEA) on premolar extraction orthodontic models and found that tooth movement affected stress distribution in the maxilla. Wolff’s Law [9] in biomechanics states that changes in the force applied to bones can cause changes in their internal structure and external morphology, such as bone density and thickness variations. During orthodontic treatment involving premolar extraction, the initial position and force distribution of the zygomatic pillar change after applying corrective forces to the first molars. Based on the primary function of the zygomatic pillar in bearing the occlusal forces of the first molars, we hypothesized that zygomatic pillar remodeling might occur. An immediate decrease in total occlusal force was observed in patients after orthodontic treatment involving premolar extraction [10, 11]; thus, we further speculated that this phenomenon might be attributed to pillar remodeling.

To determine the effects of orthodontic molar movement on the morphology and structure of the zygomatic pillar, to investigate how 3D movements of the maxillary first molar result in zygomatic pillar remodeling, and to explore the potential effect of this structural remodeling on post-orthodontic stability, we designed methods to evaluate zygomatic pillar remodeling. Person correlation studies were performed and linear regression equations were used to determine the effects of the 3D movements of the maxillary first molar on zygomatic pillar remodeling. Subsequently, 3D FEA was performed to analyze the distribution of occlusal forces on the zygomatic pillar, thereby explaining changes in occlusal forces and bite contact characteristics immediately after the completion of orthodontic treatment as shown by Teetester. The clinical demands addressed herein guide for controlling the 3D direction of molar movement clinically, starting from supporting structural remodeling. Additionally, by speculating the potential effect of zygomatic pillar remodeling on occlusal stability, the present study provides a basis to investigate the reasons behind orthodontic relapse.

## Materials and methods

### Subjects

Data on alveolar buccal bone thicknesses [12], bone density [13], and occlusal force [10] from previous studies on orthodontic treatment were used to calculate the sample size by Gpower. The occlusal force before and after the treatment were 462.8 ± 37.8 and 229.5 ± 25.5, respectively, and for bone thicknesses and density, differences in the means and standard deviations are − 0.5 ± 0.7 mm and 349.15 ± 90.79 HU, respectively (significance level = 0.05, power = 0.80). This indicated that 14 samples were needed to reject the null hypothesis with a power of 80% and a type I error of 0.05.

Twenty-five patients with malocclusion (9 males and 16 females, with an average age of 24.5 years) treated at the Affiliated Stomatology Hospital of Nanjing Medical University from 2020 to 2021 were selected. The study was approved by the Bioethics Committee of the Affiliated Stomatology Hospital of Nanjing Medical University (Approval No. JX-2022-NL05). Inclusion criteria were as follows: (1) age, 18–35years old, (2) no history of orthodontic treatment, (3) diagnosed with skeletal Class I, Angle’s Class I, (4) average mandibular plane angle, (5) dental crowding or anterior protrusion, requiring more than 10 mm for aligning and leveling and intrusion of anterior teeth, (6) aside from the third molars, complete permanent dentition (no missing teeth, retained primary teeth, supernumerary teeth, or malformed teeth), healthy tooth structure, and periodontal tissues, (7) adopted the orthodontic treatment strategy of extracting four first premolars, (8) essentially symmetrical jawbone development, and (9) not designing TAD (temporary anchorage device)solutions such as micro-implants. Exclusion criteria were as follows: (1) patients receiving orthodontic–orthognathic combination treatment, (2) a history of maxillofacial trauma, cyst, tumor, surgery, or cleft lip and palate, and (3) a history of periodontal disease, systemic disease, temporomandibular joint disorder, or diseases affecting bone metabolism and patients consuming medications for the mentioned diseases.

All patients underwent the treatment using Damon 3MX standard torque brackets (Ormco, Glendora, CA) and buccal tubes on molars, following a specific archwire sequence given below: The maxillary teeth were bonded and aligned using nickel-titanium archwires with a sequence of 0.012’’, 0.014’’, 0.016’’, and 0.016’’ * 0.022’’ 0.018’’ * 0.025’’, followed by the use of stainless-steel archwires with a sequence of 0.018’’ * 0.025’’ as the main archwire. Subsequently, the maxillary canines underwent distalization. Molar anchorage was designed as follows: The second molar was included in the treatment, and after 6–8 months of adequate alignment and leveling, the second molar was prepared for anchorage at an angle of 10–15°. The posterior teeth were tied together, and a two-step sliding method was used to close the extraction space, in some cases, an intermaxillary traction force of approximately 150 g was applied. The average treatment duration was 2.19 ± 0.75 years. The specific explanations of certain abbreviations mentioned in the text are provided in Table 1.

**Table 1:** Definitions of reference planes variables and their abbreviations

Reference plane/Measurement	Abbreviation	Description
Frankfurt Horizontal Plane	FH plane	A plane that passes through two points: the right and left Porion and the right and left Orbitale
Infraorbital foramen Plane	IF plane	A plane that passes through the infraorbital foramen parallel to the mid-sagittal plane
Infraorbital Plane	Or plane	A plane that passes through the infraorbital point (Or) parallel to the coronal plane
Temporal zygomatic suture Plane	TZS plane	A plane that passes through the highest point of the temporal zygomatic suture (TZS) parallel to the coronal
Frontozygomatic suture Plane Maxillary first molar Plane	Fmt plane M1 plane	A plane that passes through the lowest point of the frontozygomatic suture parallel to the FH plane A plane that passes through the midpoint of the buccal alveolar crest of the maxillary first molar before treatment parallel to the FH plane
Oramen magnum Plane	DFM plane	A plane passes through the posterior margin of the foramen magnum is the DFM plane
The cross-sectional area of the zygomatic pillar	h1-h7 area	Cross-sectional area in seven height planes within the measurement range of the zygomatic pillar
Morphological landmarks on the surface of the zygomatic pillar	ZFF	Zygomaticofacial foramina
	ZP	Zygomatic prominence point (The most outward-projecting point on the body of the zygoma)
Buccal alveolar bone thickness Buccal alveolar bone density	ZM	Zygomaxillare point (The lowest point of the zygomatico-mandibular suture)
	EKM	Buccal bony eminence at the root apex of the mesiobuccal root of the first molar
	MBT1-MBT3 DBT1-DBT3	The buccal alveolar bone thickness of mesiobuccal/distal buccal root measured between the facial aspect of the root to the facial aspect of the alveolar bone at H1-H8
	MBbmd1-MBbmd3 DBbmd1-DBbmd3	The average buccal alveolar bone density of buccal alveolar bone mentioned above
Cortical bone thickness	MBT4-MBT8 DBT4-DBT8	Cortical bone thickness measured between the facial surface and the inner surface of the Cortical bone
Cortical bone density	MBbmd4-MBbmd8 DBbmd4-DBbmd8	The average buccal alveolar bone density of Cortical bone mentioned above
Total occclusal force	TOF	Total occlusal force throughout the entire dental arch
Total area of occlusal contact	TCA	Total occlusal contact area throughout the entire dental arch
Occlusal force per unit area	TOF/TCA	Occlusal force per unit area assumed
First molar OF	First molar OF	Occlusal force of bilateral first maxillary molar
Bilateral measurement	△bi-	Total deference of bilateral body measurement for items between Timepoint T1 and T0
Occlusal force ratio of	OF ratio of	The sum of the occlusal forces of the tooth in the segment as a percentage of the total occlusal force

### Cone-beam computed tomography (CBCT)

#### Radiographic analysis

**Fig. 1:**
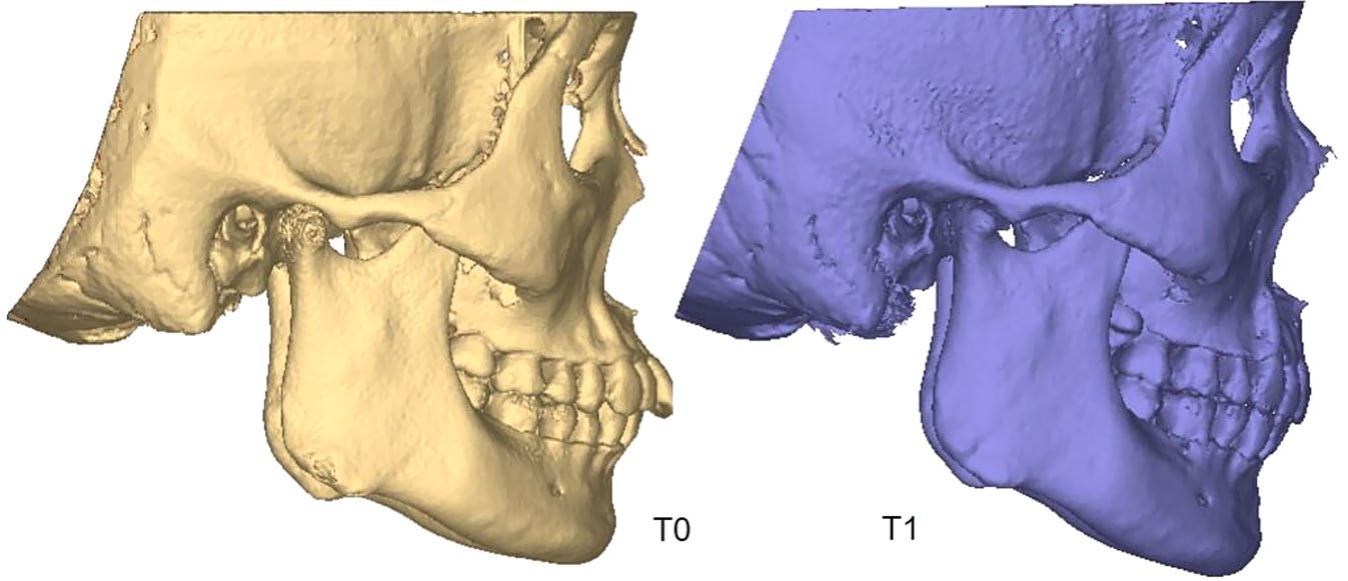
Three-dimensional models of craniomaxillofacial bone before and after the extraction orthodontic treatment (T0, T1)

CBCT images (NewTom VGi [QRsrl, Verona, Italy]) were taken before the treatment (T0) and on the day of orthodontic appliance removal (T1). The resulting images were imported into Mimics Medical 21.0, the 3D measurement software (Materialise, Belgium). The orbital–ear, mid–sagittal, and coronal planes perpendicular to both planes were used as 3D reference planes. Pretreatment (T0) and post-treatment (T1) craniofacial bone and teeth 3D images were extracted, and landmark localization and index measurement were performed (Fig. 1). Detailed operations are as follows:

#### Zygomatic pillar measurement range

**Fig. 2:**
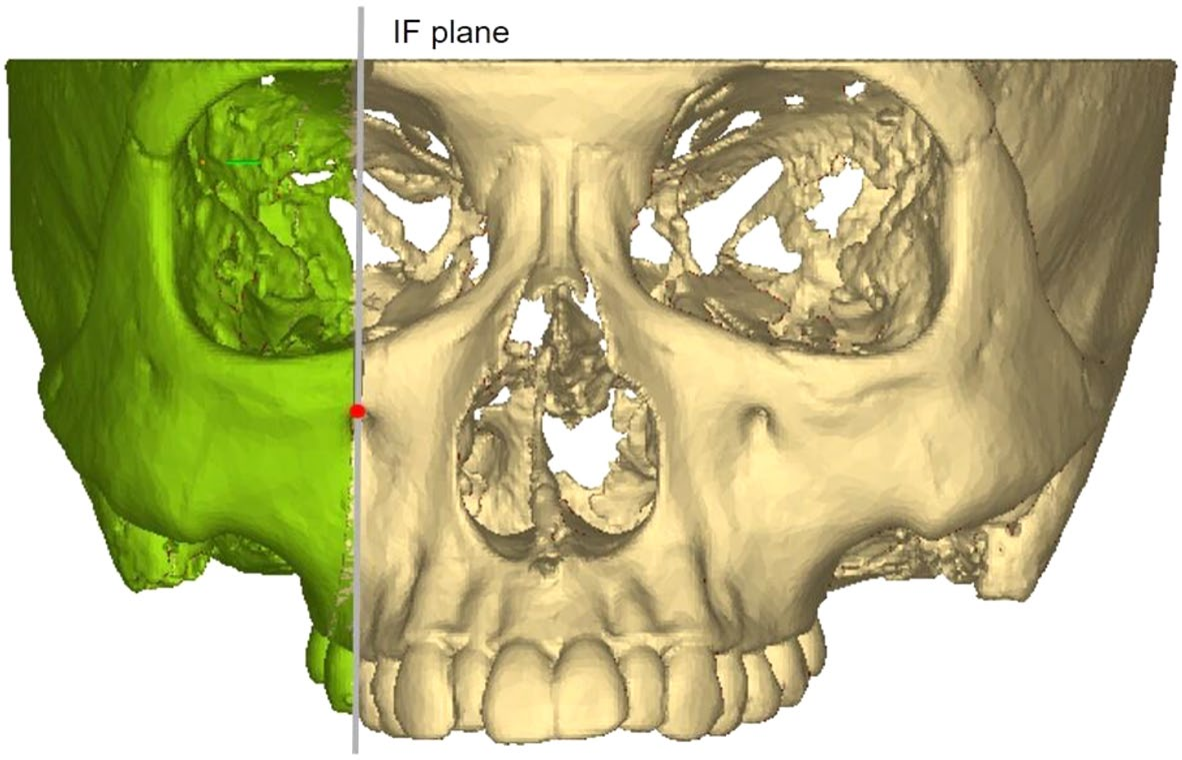
Transverse measurement range of zygomatic pillar

**Fig. 3:**
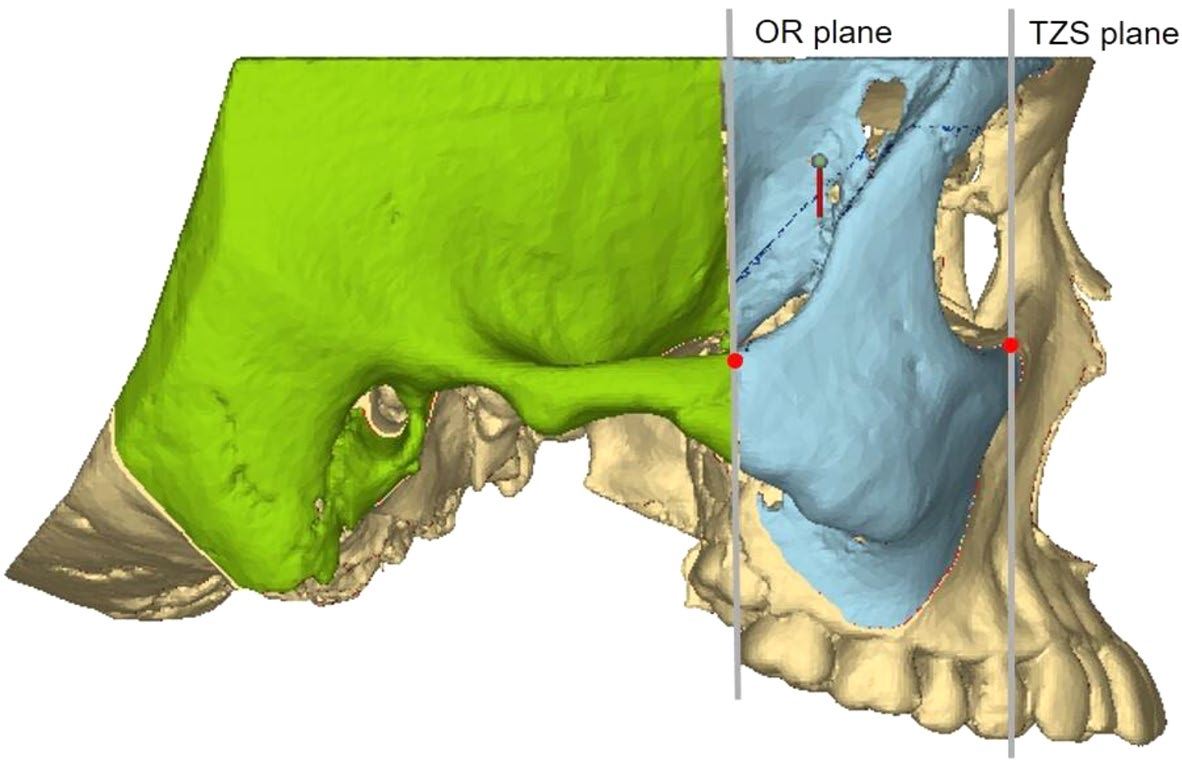
Sagittal measurement range of zygomatic pillar

**Fig. 4:**
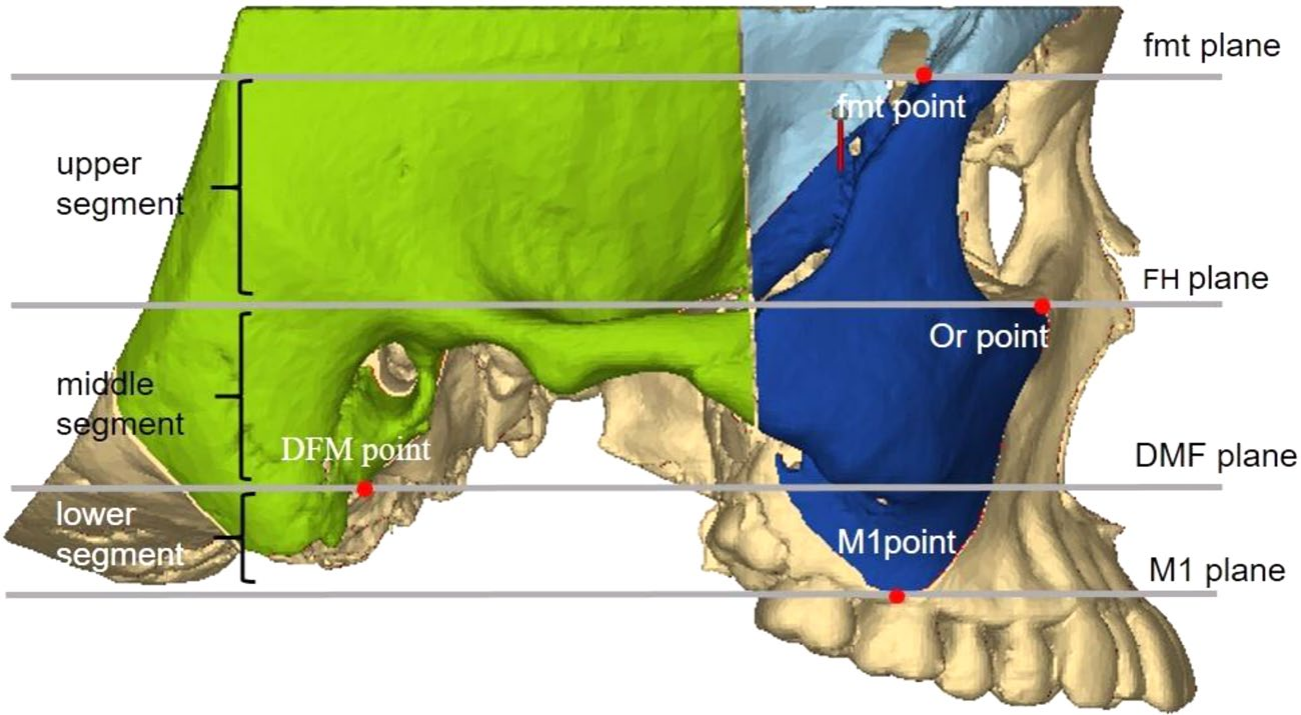
Sagittal measurement range of zygomatic pillar

**Fig. 5:**
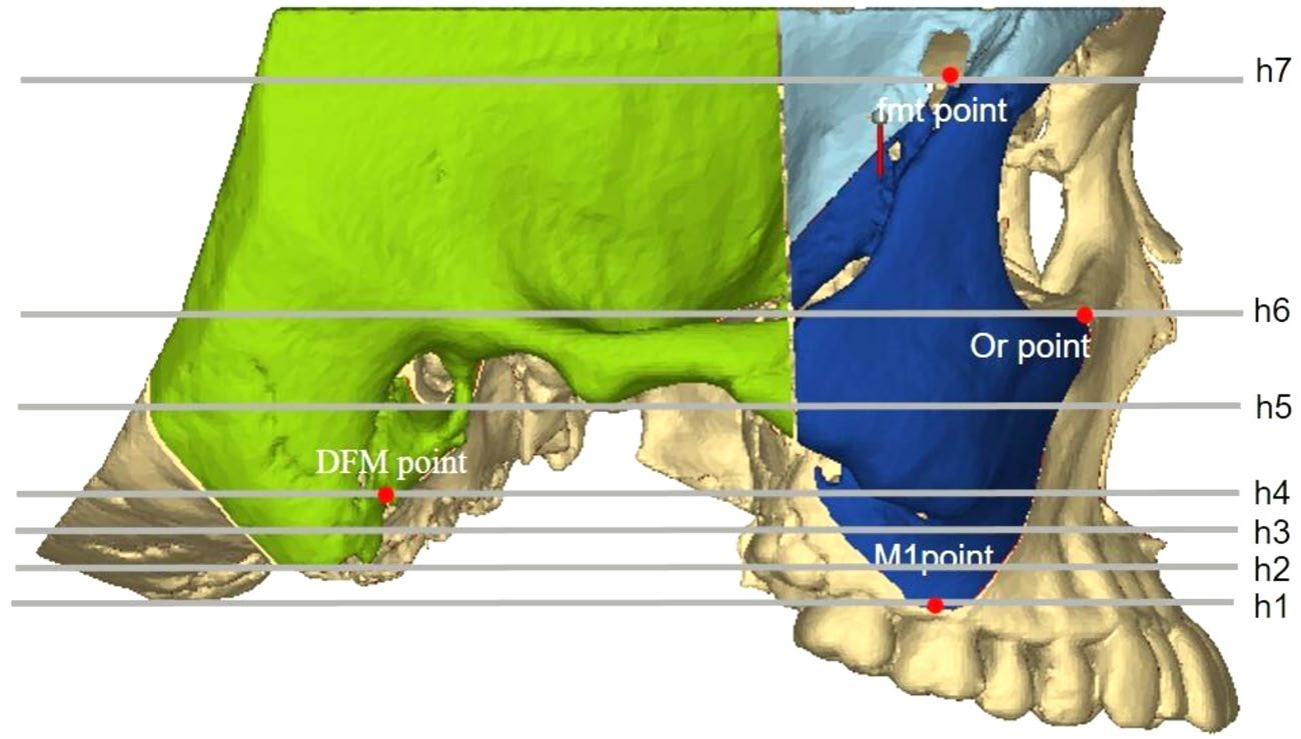
Seven stable height planes

To accurately analyze changes in the zygomatic pillar after orthodontic tooth movement, external reference lines, constructed from stable skeletal structures, were used to define the range. (1) Coronal range (horizontal): between the IF plane and the buccal lateral margin (Fig. 2). (2) Sagittal range: between the OR plane and the TSZ plane (Fig. 3). (3) Vertical range: between the FMT plane and the M1 plane. The FMT, FH, and DMF planes divided the vertical range of the zygomatic pillar into upper, middle, and lower segments, respectively (Fig. 4). Changes in the morphology of the zygomatic pillar were assessed. The cross-sectional area of the zygomatic pillar was measured as follows: The lower segment was divided into thirds and the middle segment into halves, resulting in seven height planes, namely h1–h7 (Fig. 5).

#### Changes in zygomatic pillar morphology

**Fig. 6:**
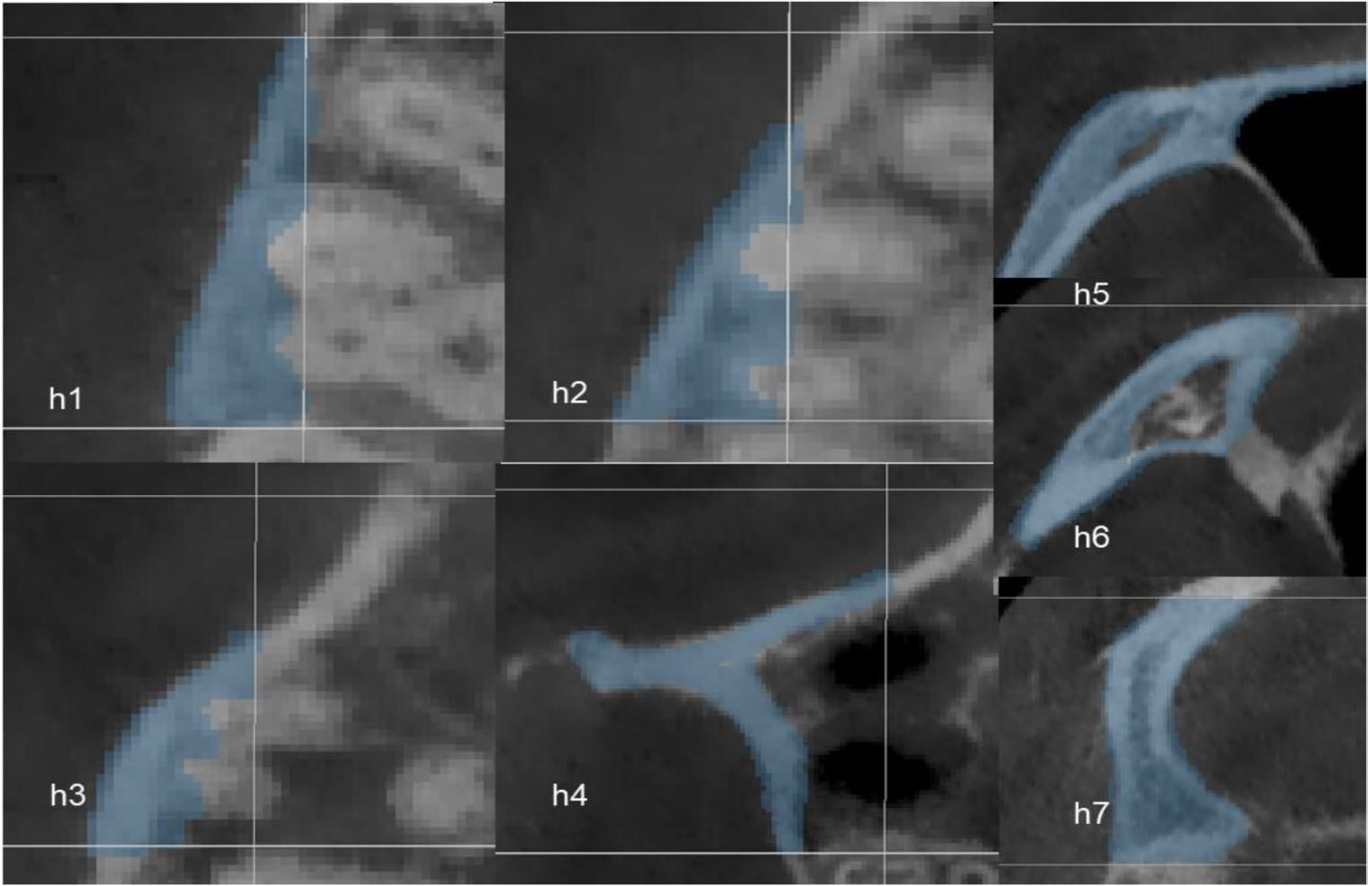
Cross-sectional area of zygomatic pillars in seven height planes (h1–h7)

**Fig. 7:**
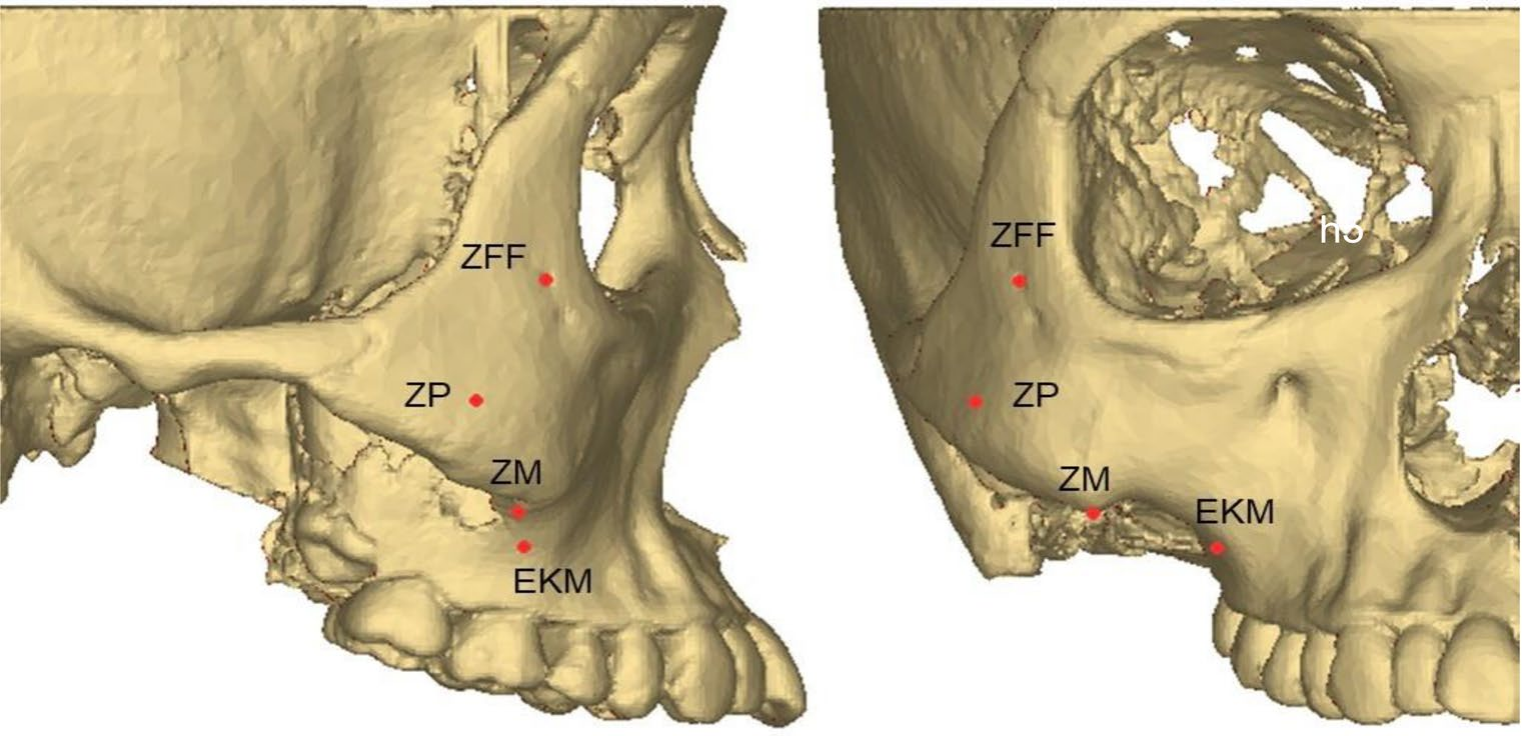
Zygomatic pillar surface morphology marker Points

**Fig. 8:**
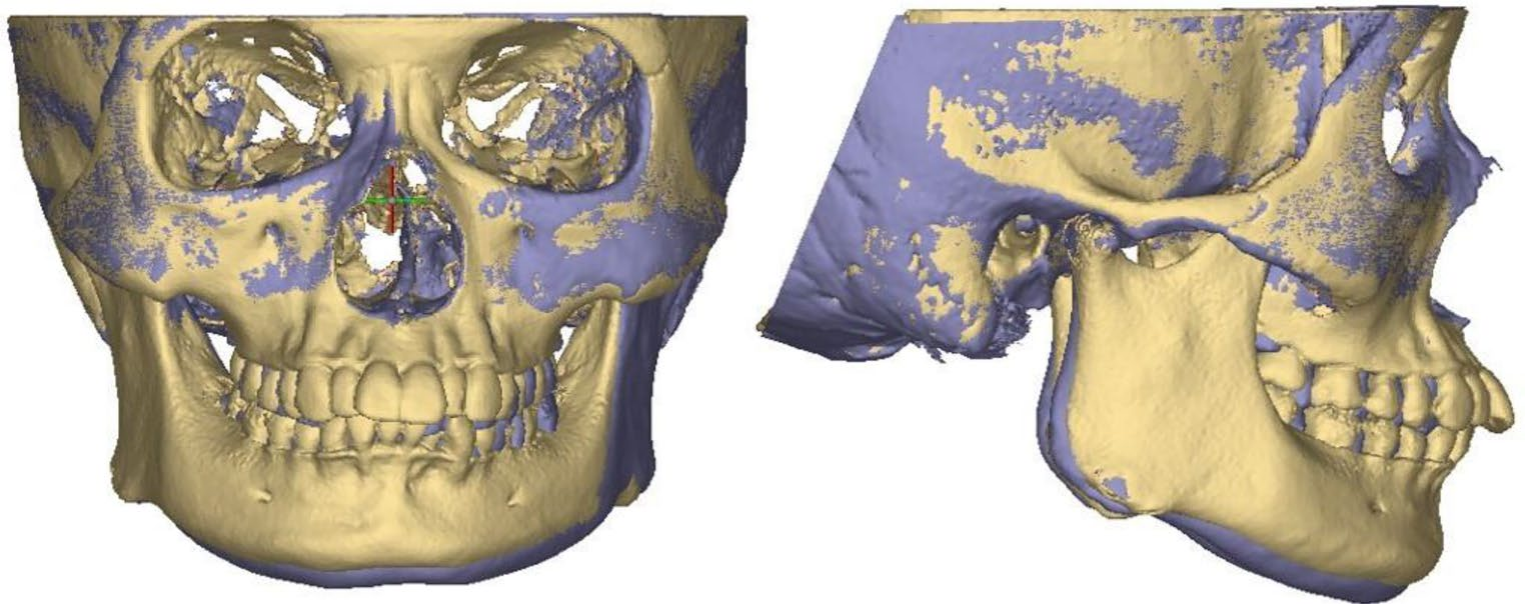
3D models overlapped before and after the extraction orthodontic treatment

Changes in the cross-sectional area in the seven stable height planes were measured within the fixed pillar range before and after the treatment to reflect changes in the overall morphology of the zygomatic pillar (Fig. 6). The surface morphology landmark of the zygomatic pillar was analyzed as follows: The following four landmarks were located in the zygomatic pillar area: △ZFF, △ZP, △ZM, and △EKM (Fig. 7). Using Mimics, the pretreatment and post-treatment 3D models of the same patient were overlaid to analyze changes in the coordinates of the landmarks using the coordinate system of the software, thereby reflecting changes in the surface morphology of the zygomatic pillar (Fig. 8).

#### Structural changes in the zygomatic pillar

**Fig. 9:**
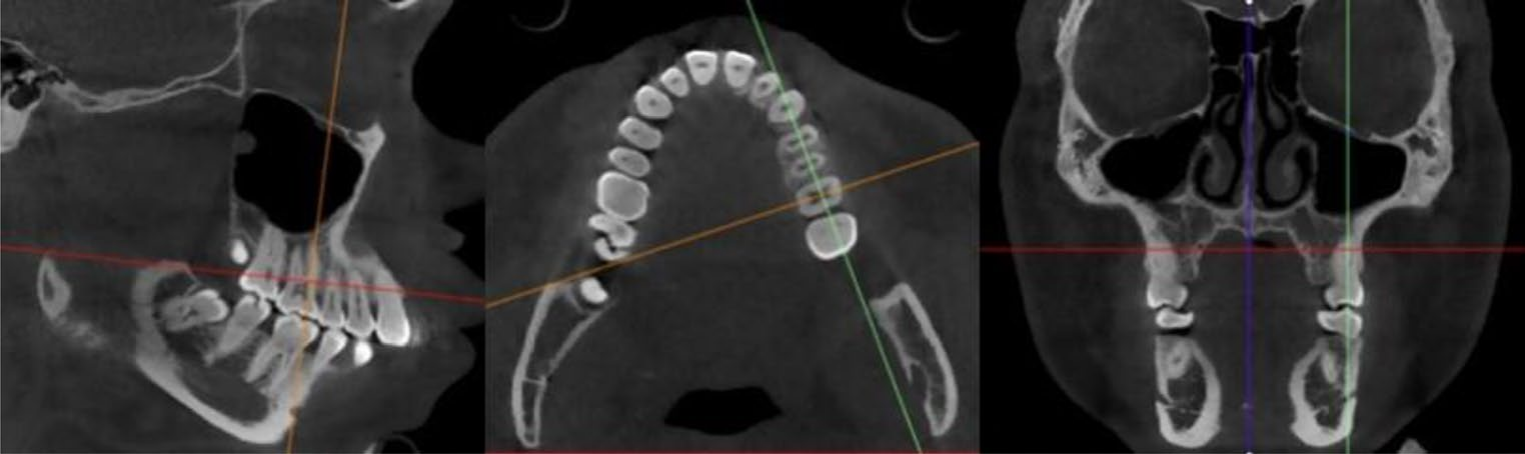
Three views of CBCT. (a) sagittal view, (b) cross-sectional, (c) axial slices

**Fig. 10:**
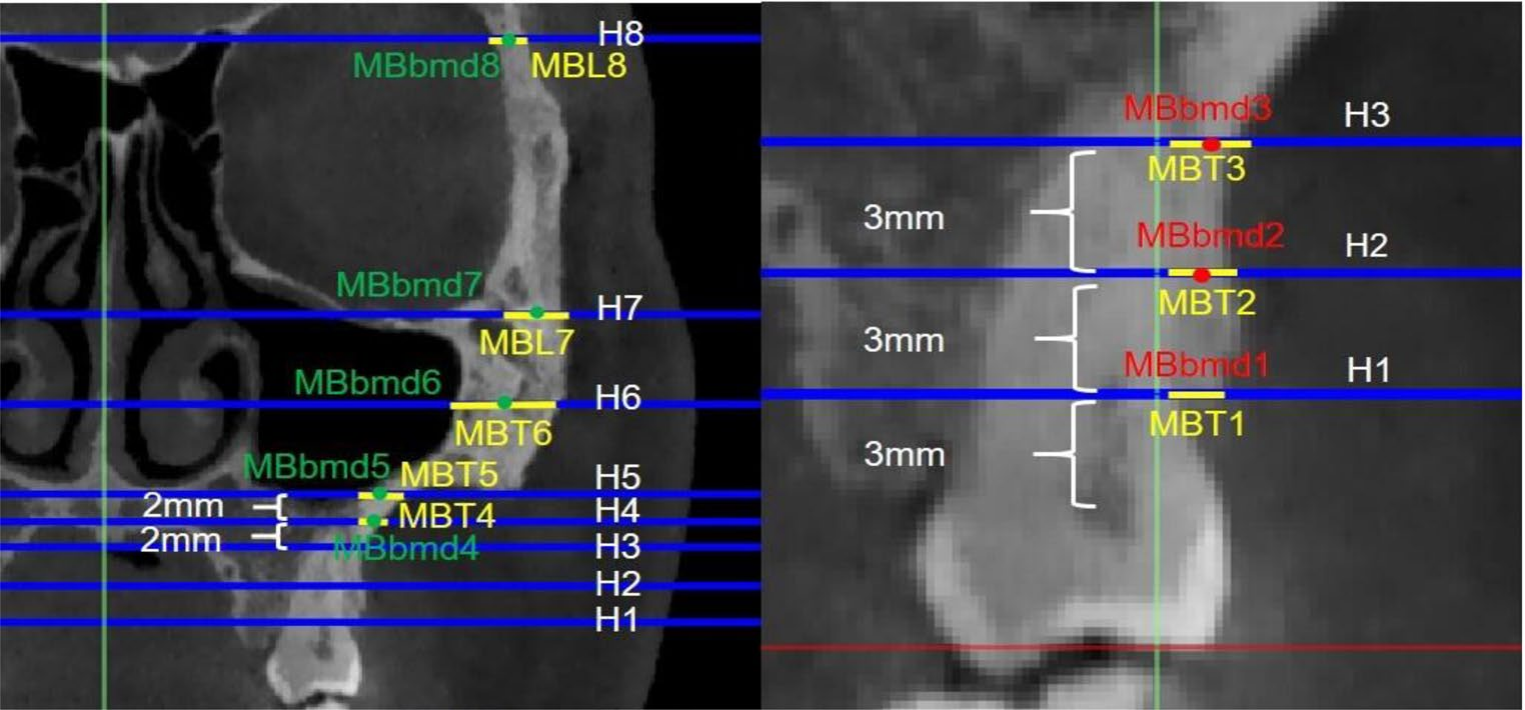
Measurement of the bone thickness and bone mineral density of alveolar bone of maxillary first molar and cortical bone of zygomatic pillar (red ellipse: alveolar bone density; green ellipse: cortical bone density; yellow line: bone thickness)

The bone thickness and density of the zygomatic pillar were measured. Based on Juliana F’s study [12], the 3D window in the Mimics software was adjusted (Fig. 6) to set axial slices and cross sections (Fig. 9). In the axial slices of the mesiobuccal and distobuccal root long axes of the maxillary first molar, the following variables were assessed: Alveolar bone area: At 3 mm, 6 mm, and 9 mm above the CEJ (heights, h1–h3), buccal alveolar bone thickness and buccal alveolar bone density were measured in Hounsfield units by creating a 1.0-mm² ellipse in the bone using Mimics (Fig. 10). Cortical bone area: Heights h4 and h5 were defined at 2 mm and 4 mm above the root apex, respectively. h6–h8 were similar to h5–h7. Cortical bone thickness and density were measured using the same method [13].

#### Maxillary first molar 3D movement and rotation

Based on the methods of Dai FF et al. [14], we established a coordinate system using a constant reference plane to measure changes in the mesial–distal, buccolingual, and vertical movement distances and mesial–distal and buccolingual angulation of the maxillary first molar.

### Occlusal characteristics and occlusal stress distribution analysis

**Fig. 11:**
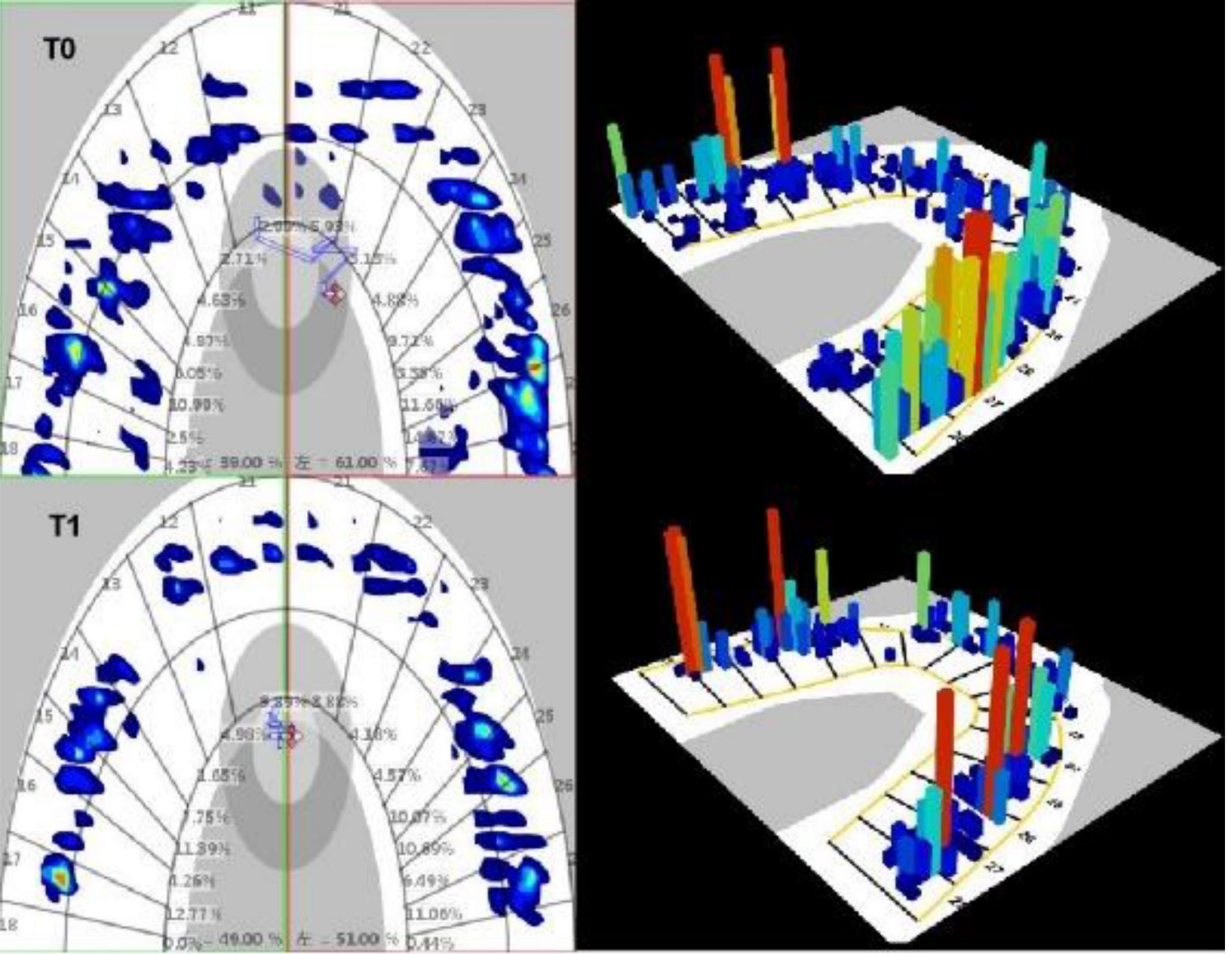
Teetester analysis images at T0 and T1

We then performed an occlusal analysis using Teetester 3.4 Occlusal Analysis System (E-Motion Inc., China) (Fig. 11). The patient was seated with the FH plane parallel to the floor, and the sensor was placed gently inside the patient’s mouth. Occlusal load characteristics were recorded from the rest position to the intercuspal position (ICP). The process was repeated thrice. For the measurements, when the total force of ICP reached 100%, the following data were recorded: TCA, TOF, TOF/TCA, OF ratio of the first molar, OF ratio of anterior teeth, OF ratio of premolar (only bilateral second premolars were recorded after the treatment), and OF ratio of the molar.

Unlike Dong-Soon Choi’s study [7], we selected pretreatment and post-treatment CBCT data from a 23-year-old female patient to produce two 3D FE models of a human skull (T0, T1). Then, we applied the average combined forces obtained from the experiments before and after the treatment on each tooth in both models to maximally simulate the stress distribution of occlusal forces on the craniofacial bones before and after zygomatic pillar remodeling.

### Statistical analyses

We performed statistical analyses using SPSS 26.0. Radiographic data were measured twice by the same doctor after 2 weeks to evaluate measurement errors and reliability. Repeated measurements were assessed using the paired t-test (systematic errors) and the Dahlberg formula (casual errors) [15]. No significant systematic errors were found (*P* > 0.1), and the random errors were small, showing high rates of reproducibility. Intraobserver reliability was assessed by calculating the intraclass correlation coefficient (0.834–0.968). Thus, the average of the two measurements was considered. The t-test showed no statistically significant differences in the data for the same measurements on both sides of the patients; thus, the data from both sides of the same patient were combined for statistical analysis. The Shapiro–Wilks test was used to test whether the variables were normally distributed. Levene’s test was used to calculate the equality of the variance of the data with normal distribution before and after the treatment. The results showed that the p-value of each group was greater than 0.05, indicating that the variance was homogeneous. Then paired-sample t-test (^t^) or Wilcoxon rank-sum test (^w^) was used to compare the differences in each index before and after the treatment.

Pearson’s correlation analysis was performed to assess the correlation between the statistically significant indices of zygomatic pillar reconstruction, maxillary first molar movements, and angular changes, as well as their correlations with changes in occlusal characteristics. A multivariable linear regression model was used to investigate the effect of maxillary first molar movements on zygomatic pillar reconstruction. The results are presented as‾x ± s or P50 (P25, P75)^w^. The P-value < 0.05 was considered statistically significant.

## Results

### Remodeling of zygomatic pillar morphology

The overall cross-sectional area decreased after the treatment (Table 2). Specifically, the decrease in the cross-sectional area at the three height planes of the lower segment of the zygomatic pillar was statistically significant (*P* < 0.05), with more significant changes observed at the two height planes closer to the alveolar crest. The overall morphology of the lower part of the zygomatic pillar was reduced, and the reduction in the base pillar shape beneath the tooth crown, where orthodontic and occlusal forces act, was more significant (*P* < 0.001).

**Table 2:** Comparison of the cross-sectional area measurements at 7 heights of the zygomatic pillar at T0 and T1

Area	T0	T1	T0-T1	Z/t	*P*
h1a	29.81 ± 5.39	27.48 ± 5.91	1.64 ± 4.99	4.182	0.000^**^
h2a	43.61 ± 6.11	40.96 ± 5.35	2.66 ± 4.14	4.530	0.000^**^
h3a	62.55 ± 5.40	60.77 ± 6.29	1.78 ± 5.37	2.348	0.023^*^
h4a	61.53 ± 20.21	62.71 ± 18.53	1.18 ± 8.06	-1.04	0.305
h5a	162.17 ± 36.06	162.09 ± 34.34	0.09 ± 8.52	0.073	0.942
h6a	302.33 ± 70.55	300.90 ± 65.72	1.43 ± 28.54	0.353	0.725
h7a	121.35 ± 38.79	118.05 ± 38.84	3.30 ± 16.72	1.39	0.170

^*^P < 0.05, ^**^P < 0.01

The coordinates of the surface morphological landmarks ZP on the X and Y axes and ZM on the Y axis decreased significantly, indicating that the ZP point moved inward and backward, whereas the ZM point moved backward (Table 3). The EKM point was significantly displaced with the movement of the maxillary first molar, shifting mesially, palatally, and downward. Thus, after reduction orthodontics, the mid-section surface morphological landmarks (ZM and ZP) of the zygomatic pillar moved backward, the ZP point moved inward, the pillar shape at this location shifted backward, and the starting point of the pillar moved forward.

**Table 3:** Comparison of the zygomatic pillar landmarks at T0 and T1

Measurement		T0	T1	T0-T1	t	*P*
ZFF	X	50.53 ± 3.89	50.83 ± 3.71	0.30 ± 1.97	-1.08	0.285
	Y	32.50 ± 5.87	32.20 ± 6.12	0.30 ± 1.97	-1.90	0.064
	Z	16.59 ± 3.02	16.71 ± 3.33	0.12 ± 1.89	0.452	0.653
ZP	X	54.93 ± 4.93	54.51 ± 4.74	0.42 ± 0.62	4.757	0.000^**^
	Y	30.37 ± 6.10	29.06 ± 6.63	1.31 ± 1.54	-6.095	0.000^**^
	Z	16.61 ± 2.92	16.74 ± 3.30	0.12 ± 1.93	-0.439	0.662
	X	47.53 ± 5.34	47.64 ± 5.1	0.11 ± 1.5	-0.528	0.600
ZM	Y	31.17 ± 5.50	29.67 ± 5.69	1.51 ± 1.39	-7.678	0.000^**^
	Z	-7.74 ± 2.36	-7.66 ± 2.51	0.80 ± 1.24	-0.459	0.649
EKM	X	30.07 ± 3.93	29.24 ± 3.92	0.83 ± 1.26	4.666	0.000^**^
	Y	32.25 ± 4.70	33.65 ± 4.66	-1.40 ± 0.488	20.258	0.000^**^
	Z	-14.36 ± 1.68	-14.94 ± 1.66	0.58 ± 0.63	6.52	0.000^**^

^*^P < 0.05, ^**^P < 0.01

### Remodeling of the zygomatic pillar structure

Consistent with the changes in the cross-sectional area of the pillar, both the alveolar bone thickness and bone density of the maxillary first molar in the zygomatic pillar region decreased (Table 4). Compared with the apical 1/3rd location, i.e., 9 mm above the CEJ, reconstruction at locations 3 mm and 6 mm above the CEJ were more significant (except MBT3) (*P* < 0.001).

Most cases had no bone structure at h9 of the coronal plane of the tooth axis, h9 data were excluded. The cortical bone thickness of the zygomatic pillar decreased significantly except for h8; however, no statistically significant difference was observed for cortical bone density (Tables 5 and 6).

**Table 4:** Comparison of the thickness and density of buccal alveolar bone of maxillary first molar at T0 and T1

Measurement		T0	T1	T0-T1	t	*P*
MB	H1BT	1.65 ± 0.78	1.45 ± 0.81	0.20 ± 0.65	2.201	0.033
	H2BT	1.77 ± 0.80	1.25 ± 0.76	0.27 ± 0.68	6.404	0.000^**^
	H3BT	3.37 ± 1.68	2.69.37	0.68 ± 1.00	4.825	0.000^**^
DB	H1BT	2.49 ± 0.82	2.23 ± 0.78	0.26 ± 0.47	3.966	0.000^**^
	H2BT	2.29 ± 0.88	2.01 ± 0.80	0.28 ± 0.47	4.235	0.000^**^
	H3BT	3.55 ± 1.60	3.10 ± 1.05	0.73 ± 1.42	2.431	0.019^**^
MB	H1Bbmd	1015.26 ± 193.61	863.28 ± 155.44	152.09 ± 225.38	4.772	0.000^**^
	H2Bbmd	1109.85 ± 225.51	986.48 ± 211.89	123.37 ± 166.03	5.254	0.000^*^
	H3Bbmd	1114.84 ± 122.56	1065.45 ± 118.38	49.36 ± 81.78	4.271	0.000^**^
DB	H1Bbmd	922.53 ± 144.52	807.95 ± 182.47	44.58 ± 157.34	5.149	0.000^**^
	H2Bbmd	975.82 ± 188.32	884.67 ± 194.21	4.26 ± 83.57	7.795	0.000^**^
	H3Bbmd	1115.88 ± 123.62	1084.21 ± 151.10	31.67 ± 103.45	2.165	0.035^*^

^*^P < 0.05, ^**^P < 0.01

**Table 5:** Comparison of the cortical bone thickness of the zygomatic pillar at T0 and T1

Measurement		T0	T1	T1-T0	t	*P*
MB	H4BT	3.25 ± 1.06	2.94 ± 1.06	0.30 ± 0.74	2.913	0.005^*^
	H5BT	3.53 ± 0.76	3.23 ± 1.43	0.30 ± 1.00	2.138	0.03^*^
	H6BT	3.52 ± 0.76	3.24 ± 0.80	0.28 ± 0.90	2.176	0.034
	H7BT	4.72 ± 1.27	4.26 ± 1.26	0.47 ± 0.0.91	3.631	0.001
	H8BT	5.39 ± 2.69	5.25 ± 2.39	0.14 ± 1.97	0.486	0.629
DB	H4BT	2.51 ± 1.08	2.20 ± 1.07	0.31 ± 0.81	2.706	0.009^**^
	H5BT	2.78 ± 1.37	2.45 ± 1.39	0.33 ± 0.72	3.263	0.002^**^
	H6BT	2.80 ± 0.75	2.46 ± 0.81	0.34 ± 0.84	2.873	0.006^**^
	H7BT	4.01 ± 1.27	3.57 ± 1.27	0.45 ± 0.92	3.459	0.001^**^
	H8BT	6.52 ± 3.11	6.34 ± 2.97	0.18 ± 1.21	1.084	0.284

^*^P < 0.05, ^**^P < 0.01

**Table 6:** Comparison of the cortical bone density of the zygomatic pillar at T0 and T1

Measurement		T0	T1	T0-T1	t	*P*
MB	H4bmd	1072.532 ± 275.85	1090.22 ± 225.49	-17.69 ± 194.52	-0.643	0.523
	H5bmd	1137.02 ± 250.06	1138.18 ± 226.32	-1.16 ± 184.83	-0.044	0.965
	H6bmd	1183.84 ± 267.00	1111.77 ± 248.10	72.07 ± 279.88	1.82	0.075
	H7bmd	1314.12 ± 373.96	1207.34 ± 510.54	106.79 ± 380.20	1.986	0.053
	H8bmd	1331.23 ± 318.03	1296.05 ± 350.74	35.18 ± 317.30	0.784	0.437
DB	H4bmd	977.85 ± 322.23	1058.97 ± 256.28	-81.12 ± 290.38	-1.975	0.054
	H5bmd	1111.15 ± 239.53	1129.04 ± 238.23	-17.81 ± 127.94	-0.989	0.327
	H6bmd	1096.11 ± 267.69	1101.68 ± 205.27	-5.57 ± 161.89	-0.243	0.809
	H7bmd	1372.20 ± 241.83	1382.30 ± 351.23	-10.09 ± 357.35	-0.200	0.843
	H8bmd	1424.17 ± 295.95	1370.24 ± 405.73	54.04 ± 494.04	0.774	0.443

^*^P < 0.05, ^**^P < 0.01

### Correlation between maxillary first molar position, angulation changes, and zygomatic pillar remodeling with the multivariate linear regression model

The mean mesial movement, palatal movement, extrusion distance, mesial tipping, and palatal tipping degree of maxillary first molar were 1.30 ± 0.78 mm, 0.64 ± 0.92 mm, 0.09 ± 0.17 mm, 1.11 ± 1.14°, and 0.37 ± 0.82°, respectively (Table 7). Matched-pairs T-test showed that changes in the movement and inclination were significantly different between T0 and T1.

**Table 7:** Changes in maxillary first molar displacement and angulation

Measurement	‾x ± s	*P*
Mesial movement	1.30 ± 0.78	0.001^*^
Palatal movement	0.64 ± 0.92	0.000^**^
Extrusion	0.09 ± 0.17	0.000^**^
Mesial tipping	1.11 ± 1.14	0.000^**^
Palatal tipping	0.37 ± 0.82	0.001^*^

^*^P < 0.05, ^**^P < 0.001

**Fig. 12:**
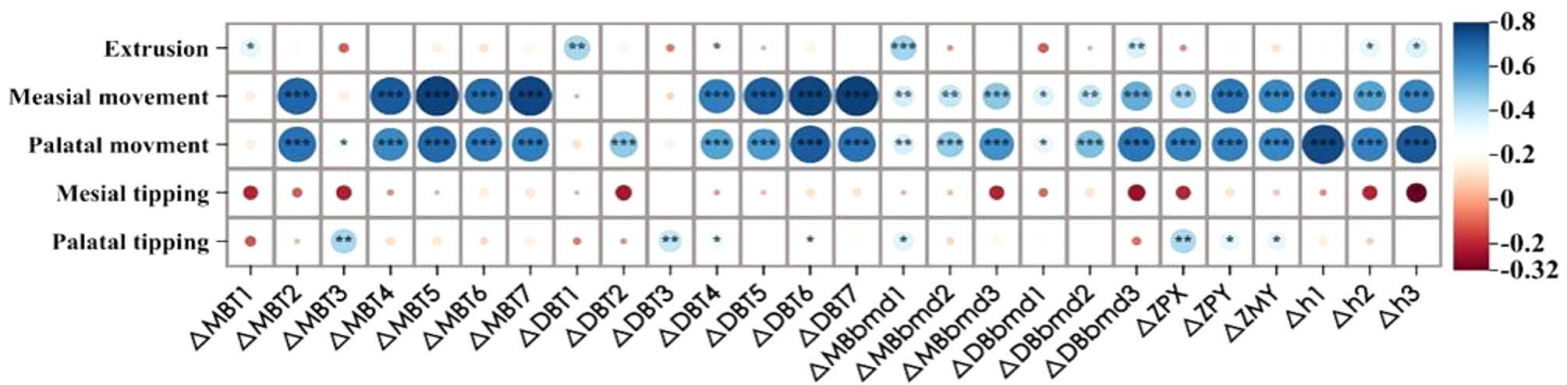
Correlation between the maxillary first molar position, angulation changes, and zygomatic pillar remodeling

Pearson’s correlation test showed a strong positive correlation between structural changes in zygomatic pillar morphology and mesiodistal and lingual movements of the molars (Fig. 12). Specifically, both △MBT4-7 and △DB4-7 showed a correlation coefficient of > 0.7 with mesial movement. △MBT1, △DBT1, △MBbmd1, and △DBbmd3 were positively correlated with extrusion, whereas △MBT3 and △DBT3 were related to palatal inclination. The backward movement of the ZP and ZM landmarks was related to mesial movement, and the inward movement of the ZP was associated with lingual movement and palatal inclination.

**Fig. 13:**
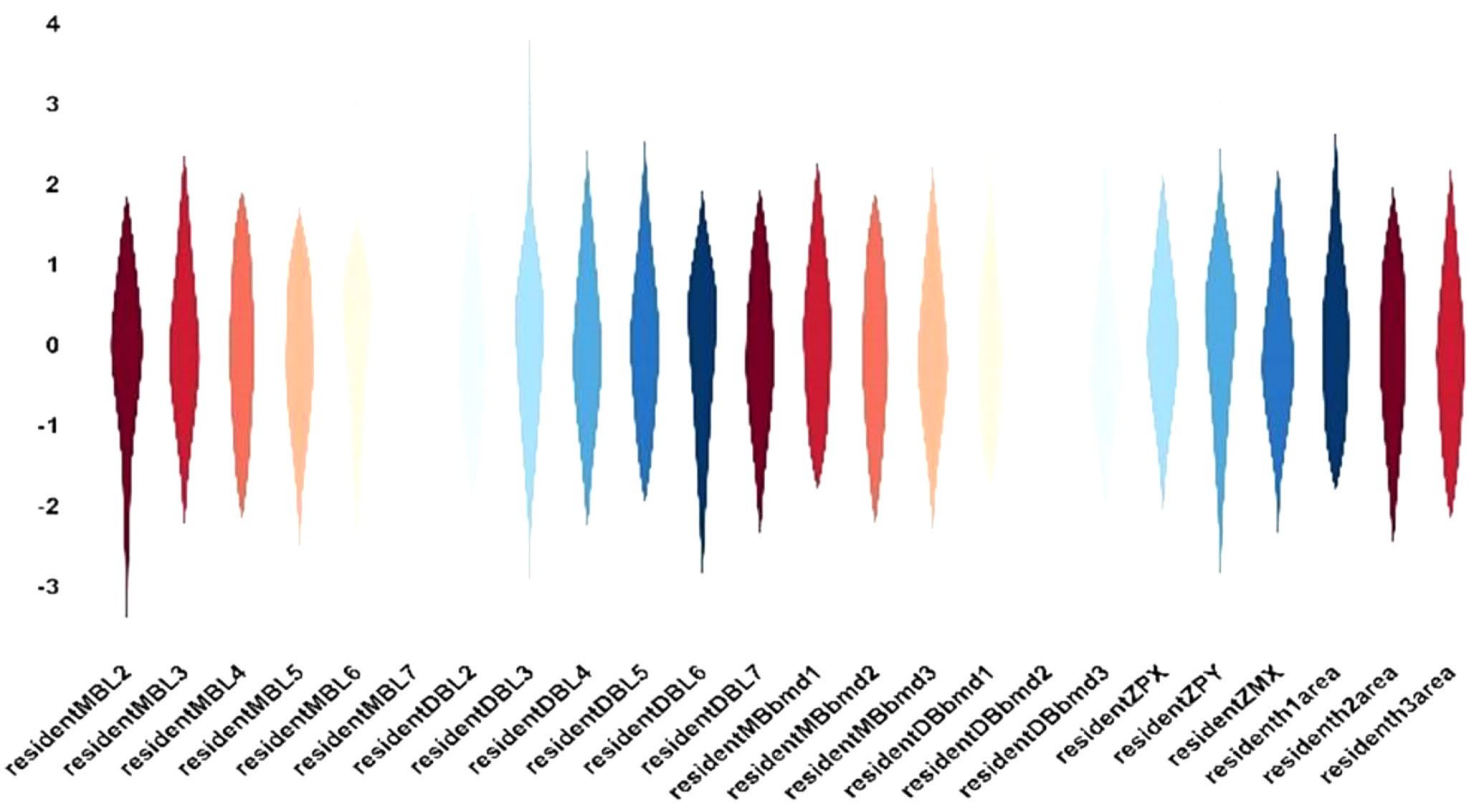
The resident distribution of the zygomatic pillar remodeling

The variables reflecting the reconstruction of pillar morphology and structure were related to the distinct types of maxillary first molar movement, based on the correlation results and the fact that dependent variables and residuals essentially follow a normal distribution without outliers (Fig. 13), we performed multiple stepwise linear regression analysis to determine the effect of changes in maxillary first molar displacement and angulation on zygomatic pillar remodeling, including only significant dependent variables (Table 8). The regression models for changes in cortical bone thickness, landmark coordinates, and cross-sectional area of the middle and upper parts of the zygomatic pillar showed adjusted R^2^ values greater than 0.4, with some values surpassing 0.6, which suggested that over 40% of the changes in cortical bone thickness, landmark coordinates, and the cross-sectional area were because of varying molar movements, indicating that maxillary first molar movement, particularly mesial and palatal movements, crucially contributes to zygomatic pillar reconstruction in patients receiving reduction orthodontic treatment.

**Table 8:** A multiple linear regression model

Response Variable	Model	*R* ^2^	Adj.*R*^2^	F	*P*
△ MBL1	0.079 + 1.374 extrusion	0.114	0.096	6.189	0.016
△ MBL2	-0.26 + 0.325 mesial movement + 0.223 palatal movement	0.561	0.543	30.066	0.000
△ MBL3	0.742 + 0.593 palatal tipping-0.254 mesial tipping	0.280	0.250	9.151	0.000
△ MBL4	-0.582 + 0.682 mesial movement	0.524	0.514	7.264	0.000
△ MBL5	-0.852 + 0.727 mesial movement + 0.333 palatal movement	0.644	0.626	42.513	0.000
△ MBL6	-0.612 + 0.521 mesial movement + 0.332 palatal movement	0.527	0.507	26.213	0.000
△ MBL7	-0.685 + 0.888 mesial movement	0.583	0.575	67.24	0.000
△ DBT1	0.144 + 1.305 extrusion	0.202	0.185	12.158	0.001
△ DBL2	0.109 + 0.249 palatal movement	0.234	0.218	14.664	0.000
△ DBL3	0.213 + 0.631 palatal movement	0.159	0.142	9.089	0.004
△ DBL4	-0.566 + 0.675 mesial movement	0.422	0.410	17.951	0.000
△ DBL5	-0.524 + 0.662 mesial movement	0.510	0.500	49.927	0.000
△ DBL6	-0.584 + 0.545 mesial movement + 0.337 palatal movement	0.651	0.636	43.799	0.000
△ DBL7	-0.697 + 0.688measial movement + 0.256 palatal movement + 0.996extrusion	0.664	0.642	30.250	0.000
△ MBbmd1	13.173 + 706.41extrusion + 102.067 palatal tipping + 57.918 mesial movement	0.445	0.409	12.295	0.000
△ MBbmd2	67.841 + 86.255 palatal movement	0.226	0.210	14.044	0.000
△ MBbmd3	14.386 + 54.378 palatal movement	0.371	0.358	28.292	0.000
△ DBbmd1	22.077 + 71.289 mesial movement	0.125	0.107	6.849	0.012
△ DBbmd2	69.730 + 51.831 palatal movement	0.258	0.242	16.654	0.000
△ DBbmd3	-8.86 + 83.674 palatal movement − 35.778 palatal tipping	0.518	0.498	22.407	0.000
△ ZPX	0.089 + 0.384 palatal movement + 0.222 palatal tipping	0.491	0.469	22.675	0.000
△ ZPY	-0.158 + 0.844 mesial movement + 0.582 palatal movement	0.503	0.482	23.790	0.000
△ ZMY	0.297 + 0.564 mesial movement + 0.656 palatal movement	0.465	0.442	20.429	0.000
△ h1area	-2.621 + 3.067 palatal movement + 1.764 mesial movement	0.611	0.595	36.937	0.000
△ h2area	0.782 + 2.911 palatal movement	0.415	0.402	23.655	0.000
△ h3area	-1.361 + 2.742 palatal movement − 1.365 palatal tipping + 2.229 mesial movement	0.636	0.612	26.603	0.000

^*^P < 0.05, ^**^P < 0.01

### Correlation of occlusal characteristics and zygomatic pillar remodeling

**Fig. 14:**
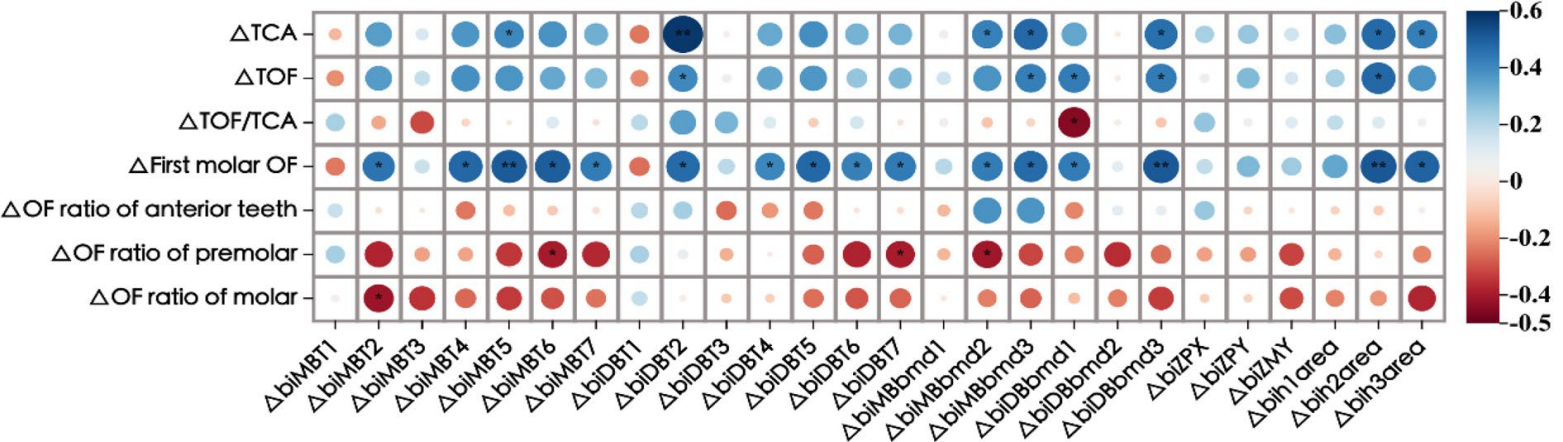
Correlation between zygomatic pillar remodeling and changes of occlusal characteristics

The total occlusal force, total occlusal area, and occlusal force of the first molar decreased after the treatment (Table 9). However, the occlusal force per unit area and the proportion of anterior teeth occlusal force increased, whereas the proportion of anterior molar occlusal force decreased. Moreover, the correlation between the thinning of cortical bone thickness in the middle and upper parts of the zygomatic pillar, reduction of alveolar bone density, and decrease in the cross-sectional area of the pillar became more apparent, with the increased occlusal force of the first molar increased more than the remaining factors (*r* > 0.4) (Fig. 14). Furthermore, the reduced alveolar bone density, except for △MBbmd1 and △DBbmd2, and decreased cross-sectional area were significantly positively correlated (*r* > 0.4), showing a decrease in the total force and total occlusal area, which indicated that zygomatic pillar reconstruction significantly affected changes in occlusal force.

**Table 9:** Changes in the occlusal characteristics

Measurement	T0	T1	Z/t	*p*
TOF (mm^2^)	342.63 ± 73.07	287.63 ± 77.31	-3.01	0.000^*^
TOA (kg)	54.56 ± 7.32	51.40 ± 9.12	-6.12	0.008^**^
TOF/TOA (N/mm^2^)	0.16 ± 0.03	0.18 ± 0.03	-5.76	0.000^**^
First molar OF (N)	14.50 ± 2.56	13.57 ± 2.76	-3.69	0.035^*^
OF ratio of first molar (%)	35 (22,31.35)	27 (22,30.5)	-0.56	0.576 (^w^)
OF ratio of anterior teeth (%)	27.68 ± 8.41	36.16 ± 6.82	-3.25	0.000^**^
OF ratio of premolar(%)	44.84 ± 9.70	17.86 ± 4.21	6.853	0.000^**^
OF ratio of molar (%)	27.00 (22.50,30.50)	45.00 (41.00, 51.50)	4.374	0.000^**^ (^w^)

^*^P < 0.05, ^**^P < 0.01

### Occlusal stress distribution in T0 and T1

**Fig. 15:**
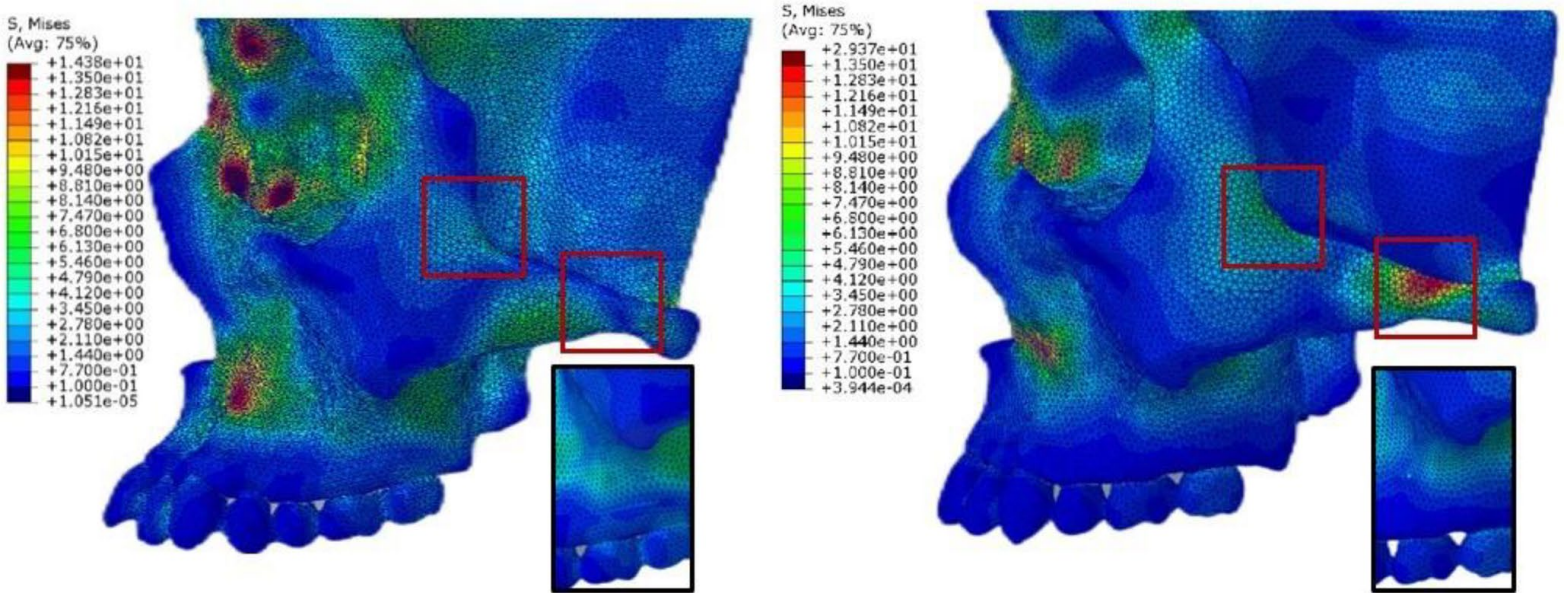
Stress distribution of the occusal force on the T0 and T1 3D FE model (black frame: stress reduction area; red frame: stress elevation area)

After zygomatic pillar reconstruction, the stress concentration area in the zygomatic alveolar ridge and buccal alveolar bone decreased, and the stress at the posterior margin of the orbit also decreased (Fig. 15). However, the stress in the zygomatic arch area behind the temporozygomatic suture and at the intersection of the vertical and horizontal edges of the zygomatic bone significantly increased.

**Discussion**.

To the best of our knowledge, this is the first quantitative study investigating zygomatic pillar remodeling resulting from 3D molar movements and angle changes during orthodontic treatment. This is the first study to use linear regression equations to determine the extent of zygomatic pillar remodeling induced by various directions and angles of the movement of the maxillary first molar within a certain range, which lays the foundation for predicting zygomatic pillar remodeling and guides in restricting the movement of the first molar in clinical practice. Previously, only associations between individual tooth movement indices and changes in alveolar bone thickness and density have been established [2, 13]. Herein, we constructed two 3D FE models of the zygomatic pillar of a patient before and after orthodontic treatment involving premolar extraction by applying the average occlusal force of the same group of patients before and after treatment. These models simulated occlusal force stress distribution before and after zygomatic pillar remodeling to the fullest extent possible, thereby showing the effect of the remodeling on occlusal characteristics and stress distribution. Furthermore, we delineated measurement areas for the zygomatic pillar and used stable skeletal landmarks as a reference to quantitatively measure the morphological and structural reconstruction of the zygomatic pillar exploratively [16–18].

Primarily, bone remodeling includes two parts, namely morphological reconstruction and structural reconstruction. Herein, the comparison of the cross-sectional areas of zygomatic pillars within a constant-height plane showed that the cross-sectional area of the lower part of the zygomatic pillar decreased significantly, thereby reflecting the overall remodeling and contraction of the lower part of the zygomatic pillar. The surface morphology landmarks of the middle section of the zygomatic pillar, namely ZM and ZP, moved backward, whereas the ZP point moved medially. This phenomenon is probably associated with the pressure on the vertical direction of the zygomaticomaxillary suture and the transversal pressure on the zygomatic body when the first molar moves forward [19]. The pressure may have led to bone resorption. Simultaneously, the EKM point moved mesially and palatally and extended along with the maxillary first molar, implying that the starting point of the zygomatic pillar moved with the maxillary first molar, whereas the middle and upper sections shifted backward and medially, respectively. Additionally, these findings may have implications for facial esthetics. The zygomatic–sphenoid suture point moves backward as the first molar moves mesially and lingually and the zygomatic point moves inward and backward. These negative changes in the bony prominences may partly explain the negative changes in the soft tissues of the cheekbone and nasolabial groove observed in some patients after orthodontic treatment [20, 21], such as the phenomenon of “brace face” in Chinese female patients with orthodontic issues. These facial changes may not be solely attributed to soft tissue remodeling but the process may involve bony prominence remodeling. Additionally, restricting the movement of the first molar may offer the possibility to reduce the effect of a “brace face.”

The alveolar bone thickness of the maxillary first molar significantly decreased; this finding is consistent with previous research involving patients who did not undergo extraction [12]. Furthermore, at all height planes, except for the FMT plane, the cortical bone thickness significantly decreased. Research using FEA may explain this phenomenon [19]. As the first molar undergoes orthodontic movement, the pressure stress area also moves forward, increasing the compression of the anterior cortical bone. Furthermore, osteoclasts are activated, resulting in bone resorption. Bone structural reconstruction, as observed in the absorption of alveolar bone density, is consistent with the findings of Chang, H [12]. Moreover, the absence of notable changes in the cortical bone density of the pillar suggests that structural reconstruction is primarily limited to the lower segment of the zygomatic pillar. In addition, the decrease in the bone thickness and density of the pillar of the zygomatic process corresponds with the observed decrease in the overall pillar shape, with the pillar surface moving inward and posteriorly.

We observed that mesial and lingual movements in reduction orthodontic treatment are most closely associated with zygomatic pillar remodeling, followed by extrusion and tipping. The MLR equation demonstrated that 40% of the changes in cortical bone thickness, coordinates, and cross-sectional areas are owing to changes in molar movement; this suggests that the movement of the maxillary first molar, particularly mesial and palatal movements, is an essential reason for zygomatic pillar remodeling in patients undergoing reduction orthodontic treatment. The results indicate that the mesial movement of the first molar is generally within approximately 2 mm, palatal movement is approximately 1.5 mm, elongation is approximately 0.2 mm, and torque and mesiodistal inclination changes are within approximately 2°. Mesial movement exerts the highest effect on pillar reconstruction, with a mesial movement of 1 mm resulting in cortical bone thickness changes of more than 0.5 mm at different heights of the zygomatic pillar. In addition, mesial movement leads to the posterior displacement of the zygomaticomaxillary suture and zygomatic process points. On the other hand, palatal movement is associated with cortical bone thinning in the upper segment of the zygomatic pillar. With palatal movement of 1 mm, the cortical bone thickness decreases by approximately 0.3 mm. Furthermore, negative torque on the molars (palatal inclination) decreases the bone density and thickness near the tooth roots in the alveolar bone. Collectively, these results reinforce the need for anchorage control, i.e., to decrease zygomatic pillar reconstruction and maintain stomatognathic system stability, we should limit the movement of the molars as much as possible. The most important strategy is limiting the mesial movement of the molars, followed by palatal and torque movements.

The occlusal force decreases after zygomatic pillar remodeling. The overall results for occlusal force are consistent with those of Yoon, W [10]. Furthermore, we observed that the occlusal force of the maxillary first molar decreased but the force ratio of the anterior teeth increased. This may be owing to the increased stress on the anterior pillar [19]. In the present study, we investigated the effect of zygomatic pillar construction on the occlusal force and observed that the more prominent the pillar bone thickness, lower segment bone density, and landmark displacement, the higher the decreasing trend of the occlusal force of the maxillary first molar. Furthermore, it affects the total occlusal force and occlusal area, corresponding to the function of the zygomatic pillar in bearing occlusal forces. After orthodontic treatment with premolar extraction, the overall structure of the zygomatic pillar becomes weaker, significantly decreasing the corresponding occlusal force that can be borne. Occlusal stress analysis of the 3D FE model after zygomatic pillar remodeling revealed a difference compared with the analysis of the simple tooth alignment model after extraction [8]. Stress is concentrated in the weak areas of the zygomatic pillar (near the most convex point of the zygomatic arch and the intersection area of the vertical and horizontal edges); this implies that the ability of these fracture-prone areas to withstand the effects of external force is further decreased, lowering the resistance of the zygomatic pillar. Zygomatic pillar remodeling may further affect the occlusal contact and stability of the mechanical distribution of the maxillofacial region, which is associated with recurrence after orthodontic treatment. Therefore, our study provides a novel idea for decreasing recurrence after orthodontic treatment: by strengthening the anchorage and restricting the movement of the molars, zygomatic pillar reconstruction can be decreased and stomatognathic system stability can be enhanced.

Our study results confirm that in patients with skeletal Class I malocclusion undergoing orthodontic treatment with extraction of the four first molars, molar movement reconstructs the morphology and structure of the zygomatic pillar. Furthermore, it quantitatively analyzes the specific effects of 3D molar movement and angle changes on zygomatic pillar reconstruction. In addition, it elucidates how these changes in the zygomatic pillar affect occlusal contact characteristics and stress distribution in the zygomatic pillar. Our research not only fills a gap in the field of pillar reconstruction but also additionally emphasizes the importance of enhancing resistance for the overall stability of the maxillofacial system. Moreover, it provides novel insights for decreasing post-orthodontic relapse and enhancing facial aesthetics by mitigating zygomatic pillar remodeling.

This study has the following shortcomings. Research on the remodeling of the entire alveolar process at different retention stages after completing orthodontic treatment was not included The stability of the zygomatic pillar during the retention period is vital for the long-term effectiveness of the orthodontic treatment because occlusal force may recover during the retention period [22]. Therefore, further expanding the sample size and extending the follow-up observation time are warranted. Moreover, owing to the clinical diversity observed, including patients with skeletal Class II and Class III malocclusions and those with varying degrees of decreased mandibular plane angles and increased vertical dimensions, the morphology and structure of the zygomatic pillar and the required level of molar movement for orthodontic correction may differ from the patients included in the present study [23]. Therefore, in subsequent experiments, we intend to investigate the effects of molar movement on zygomatic pillar reconstruction in patients with different skeletal classifications and vertical facial skeletal patterns to identify any discrepancies. Furthermore, the scope of the analysis will be expanded to include occlusal contacts in the mandibular arch and stress distribution within the mandible [24].

## Conclusions

1.In patients with Angle Class I malocclusion who underwent extraction of the four first premolars, the 3D movement of the maxillary first molar before and after orthodontic treatment resulted in zygomatic pillar remodeling.

Morphological remodeling: A decrease in the cross-sectional area of the lower segment of the zygomatic pillar and the movement of the middle and lower tuberosity points inward and backward, respectively.

Structural remodeling: Thinning of the thicknesses of the alveolar and cortical bones and a decrease in alveolar bone density.

2. Among them, the effect of mesial movement within some range on the structural remodeling (cortical bone thickness) of the zygomatic pillar was the most significant, followed by palatal movement.
3. The decrease of stress on the zygomatic alveolar crest and the increase of stress on the bend of the zygomatic bone and the suture junction may partly explain the correlation between the decrease of occlusal force and the remodeling of the zygomatic pillar.
4. Occlusal stability may be one of the reasons for orthodontic relapse, with zygomatic pillar remodeling affecting the characteristics of occlusal contact.

Therefore, these findings underscore the importance of resistance to zygomatic pillar remodeling, particularly decreasing mesial movement of the molars.

## Data Availability

No datasets were generated or analysed during the current study.
